# Supplementary material for: Immunogenicity, Safety, and Immune Persistence of One Dose of SARS-CoV-2 Recombinant Adenovirus Type-5 Vectored Vaccine in Children and Adolescents Aged 6–17 Years: An Immunobridging Trial
Source: Vaccines (Basel). 2024 Jun 19;12(6):683. doi: 10.3390/vaccines12060683 (PMC11209179; doi:10.3390/vaccines12060683)
Supplement: Supplementary file 1 [file vaccines-12-00683-s001.zip › File S1.pdf]

File S1. Inclusion and exclusion criteria.

Inclusion criteria

1. Aged 6-59 years.
2. Able to understand the content of informed consent and willing to sign the informed consent
3. Able and willing to complete all the scheduled study process during the whole study follow-up period (about 12 months).
4. Axillary temperature ≤37.0°C
5. Negative serum IgM and IgG to the SARS-CoV-2
6. Not vaccinated any type of SARS-CoV-2
7. No history of epidemiological exposure to COVID-19; No travel history to medium or high risk areas in the past 21 days;
8. General good health as established by medical history and physical examination

Exclusion Criteria

1. Family history of seizure, epilepsy, brain or mental disease
2. Participant that has an allergic history to any ingredient of vaccines
3. Woman who is pregnant, breast-feeding or positive in pregnancy test on day of enrollment, or is planning to be pregnant during the next 12 months
4. Any acute fever disease or infections or have a medical history of SARS
5. Have serious cardiovascular diseases, such as arrhythmia, conduction block, myocardial infarction, severe hypertension and not well-controlled
6. Major chronic illness, such as asthma, diabetes, or thyroid disease, and not well-controlled
7. Hereditary angioneurotic edema or acquired angioneurotic edema
8. Urticaria in last one year - Asplenia or functional asplenia
9. Asplenia or functional asplenia
10. Platelet disorder or other bleeding disorder may cause injection contraindication
11. Faint at the sight of blood or needles.
12. Prior administration of immunodepressant or corticosteroids, antianaphylaxis treatment, cytotoxic treatment in last 6 months
13. Prior administration of blood products in last 4 months
14. Prior administration of other research medicines in last 1 month
15. Prior administration of attenuated vaccine in last 1 month
16. Prior administration of subunit vaccine or inactivated vaccine in last 14 days
17. Being treated for tuberculosis
18. A history of COVID-19 infection/illness
19. Any condition that in the opinion of the investigators may interfere with the evaluation of study objectives
