# Supplementary material for: Immunogenicity, Safety, and Immune Persistence of One Dose of SARS-CoV-2 Recombinant Adenovirus Type-5 Vectored Vaccine in Children and Adolescents Aged 6–17 Years: An Immunobridging Trial
Source: Vaccines (Basel). 2024 Jun 19;12(6):683. doi: 10.3390/vaccines12060683 (PMC11209179; doi:10.3390/vaccines12060683)
Supplement: Supplementary file 1 [file vaccines-12-00683-s001.zip › Supplementary figure S1.pdf]

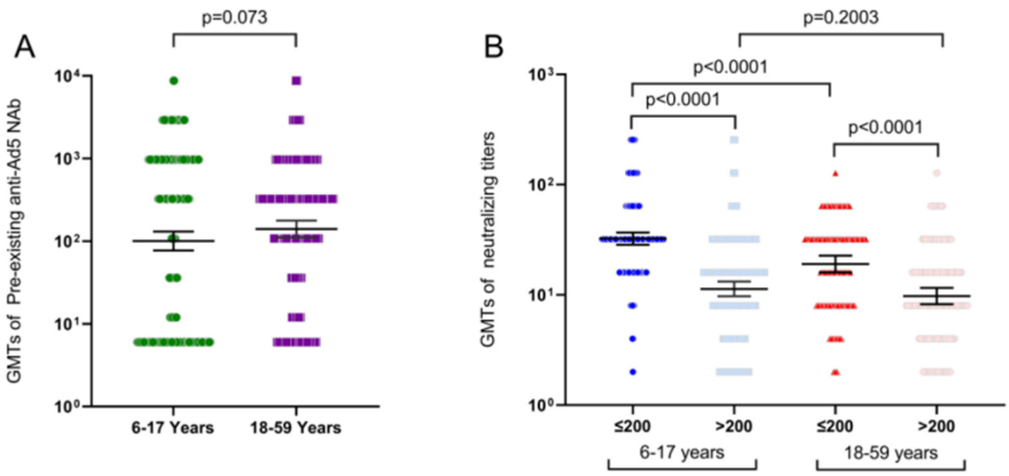

Supplementary Figure S1. A: GMTs of preexisting anti-Ad5 neutralizing antibody on day 0. B: GMTs of SARS-CoV-2 neutralizing antibodies on day 28 post-vaccination with preexisting anti-Ad5 neutralizing antibody titers ≤200 or titers ＞200.

The analysis was based on the per protocol set (PPS), included all randomized participants who received the vaccination and completed the follow-up of 28 days in accordance with the protocol. GMT = geometric mean titers. Error bars are GMT with two-sided 95% CIs.

Comparing between the two groups, the log-converted data was analyzed by T-test.
