# Supplementary material for: Immunogenicity, Safety, and Immune Persistence of One Dose of SARS-CoV-2 Recombinant Adenovirus Type-5 Vectored Vaccine in Children and Adolescents Aged 6–17 Years: An Immunobridging Trial
Source: Vaccines (Basel). 2024 Jun 19;12(6):683. doi: 10.3390/vaccines12060683 (PMC11209179; doi:10.3390/vaccines12060683)
Supplement: Supplementary file 1 [file vaccines-12-00683-s001.zip › Supplementary figure S2.pdf]

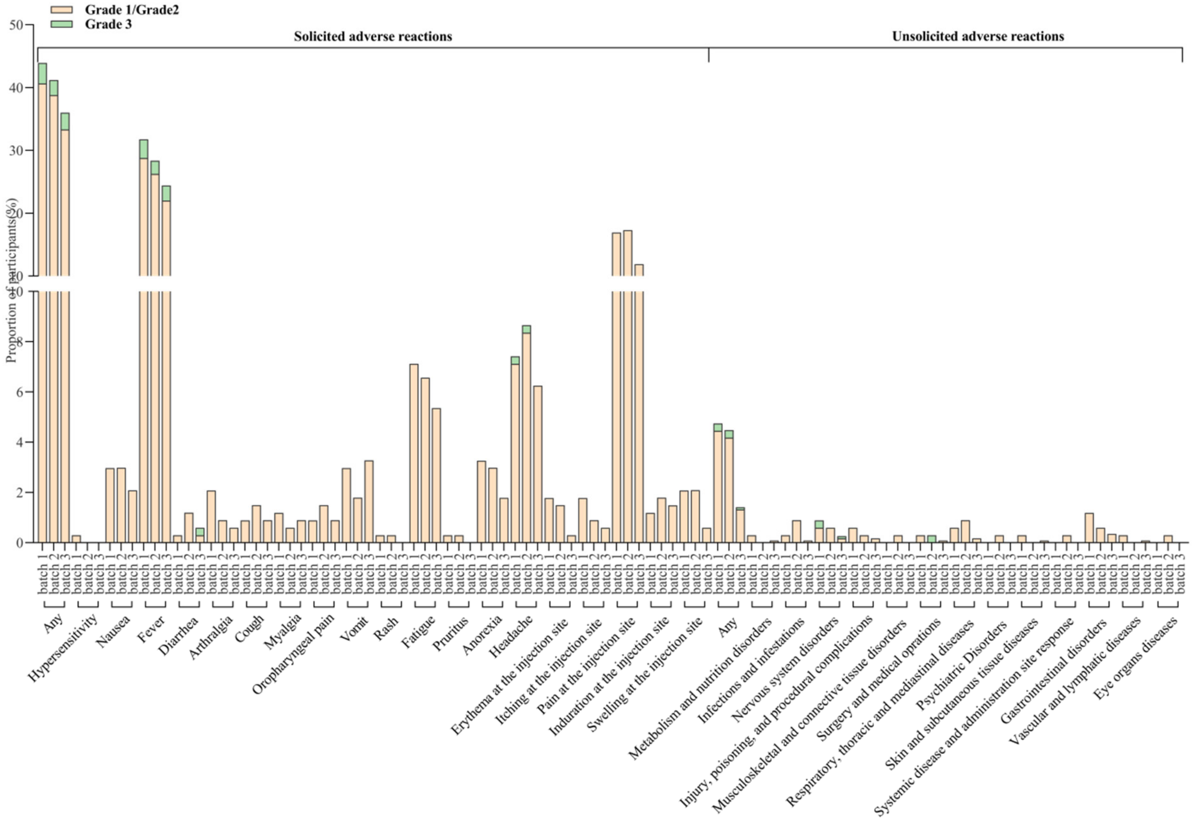

Supplementary Figure S2. Solicited and unsolicited adverse reactions occurred within 28 days after the vaccination in different batches.

Any = all the participants with any adverse reactions. The analysis was based on the safety set cohort, with all participants receiving vaccines after randomization. **P* value <0.05.
Comparisons among groups were made using the Chi-square Test or Fisher's Exact Test.
