# Supplementary material for: Immunogenicity, Safety, and Immune Persistence of One Dose of SARS-CoV-2 Recombinant Adenovirus Type-5 Vectored Vaccine in Children and Adolescents Aged 6–17 Years: An Immunobridging Trial
Source: Vaccines (Basel). 2024 Jun 19;12(6):683. doi: 10.3390/vaccines12060683 (PMC11209179; doi:10.3390/vaccines12060683)
Supplement: Supplementary file 1 [file vaccines-12-00683-s001.zip › Supplementary table S1.pdf]

Supplementary Table S1. GMTs of Neutralizing Antibodies against SARS-COV-2 (WT) on day 0 or 28 post-vaccination and subgroup by pre-existing anti-Ad5 neutralizing antibody titers. (PPS) (6-17 years old)

	800L Batch1(N=335)	800L Batch 2(N=332)	800L Batch 3(N=334)	Total(N=1001)
**Day 0**				
GMT	2	2	2	2
95% CI	2.0,2.0	2.0,2.0	2.0,2.0	2.0,2.0
**Day 28**				
GMT	18.6	17.2	19.9	18.5
95% CI	16.6,20.9	15.2,19.4	17.6,22.4	17.3,19.9
Seroconvertion N(%)	309(92.2)	302(91.0)	309(92.5)	920(91.9)
Seroconvertion rate 95%CI	88.9,94.7	87.4,93.6	89.2,94.9	90.1,93.4
**Ad5≤200**				
Day 0				
GMT	2	2	2	2
95% CI	2.0,2.0	2.0,2.0	2.0,2.0	2.0,2.0
Day 28				
GMT	32.4	33.8	33.8	33.3
95% CI	28.6,36.8	28.6,39.8	29.1,39.4	30.6,36.2
**Ad5＞200**				
Day 0				
GMT	2	2	2	2
95% CI	2.0,2.0	2.0,2.0	2.0,2.0	2.0,2.0
Day 28				
GMT	11.3	10.4	12.9	11.5
95% CI	9.7,13.2	9.0,11.9	11.0,15.1	10.5,12.5

GMT=geometric mean titer, CI: confidence interval. Seroconversion was defined as participants with detectable neutralizing antibodies if baseline titers were undetectable, or at least a four-fold increase of neutralizing antibodies from the baseline level.
