# Supplementary material for: Immunogenicity, Safety, and Immune Persistence of One Dose of SARS-CoV-2 Recombinant Adenovirus Type-5 Vectored Vaccine in Children and Adolescents Aged 6–17 Years: An Immunobridging Trial
Source: Vaccines (Basel). 2024 Jun 19;12(6):683. doi: 10.3390/vaccines12060683 (PMC11209179; doi:10.3390/vaccines12060683)
Supplement: Supplementary file 1 [file vaccines-12-00683-s001.zip › Supplementary table S2.pdf]

Supplementary Table S2. GMCs, 95%CI and seroconversion rates of SARS-CoV-2 RBD-specific IgG on day 0 or 28 post-vaccination and subgroup by pre-existing anti-Ad5 neutralizing antibody titers. (PPS) (6-17 years old).

	800L Batch1(N=335)	800L Batch 2(N=332)	800L Batch 3(N=334)	Total(N=1001)
**Day 0**				
GMC	10	10	10	10
95% CI	10.0,10.1	10.0,10.0	10.0,10.0	10.0,10.0
**Day 28**				
GMC	179.4	161.6	169.8	170.1
95% CI	159.9,201.3	143.8,181.5	151.2,190.7	159.2,181.9
Seroconvertion				
Seroconvertion N (%)	326(97.3)	320(96.4)	322(96.4)	968(96.7)
Seroconvertion rate 95%CI	95.0,98.6	93.8,97.9	93.8,97.9	95.4,97.6
**Ad5≤200**				
Day 0				
GMC	10	10	10	10
95% CI	10.0,10.0	10.0,10.0	10.0,10.0	10.0,10.0
Day 28				
GMC	345.4	333.3	307.7	328.6
95% CI	309.8,385.1	295.6,375.7	271.0,349.3	307.0,351.8
**Ad5＞200**				
Day 0				
GMC	10.1	10	10	10
95% CI	9.9,10.2	10.0,10.0	10.0,10.0	10.0,10.1
Day 28				
GMC	100	94	104.6	99.4
95% CI	86.1,116.2	81.8,108.2	89.9,121.7	91.3,108.2

GMC: geometric mean concentration, CI: confidence interval. Seroconversion was defined as participants with detectable ELISA antibodies if baseline titers were undetectable, or at least a four-fold increase of RBD-specific antibodies from the baseline level.
