# Supplementary material for: Immunogenicity, Safety, and Immune Persistence of One Dose of SARS-CoV-2 Recombinant Adenovirus Type-5 Vectored Vaccine in Children and Adolescents Aged 6–17 Years: An Immunobridging Trial
Source: Vaccines (Basel). 2024 Jun 19;12(6):683. doi: 10.3390/vaccines12060683 (PMC11209179; doi:10.3390/vaccines12060683)
Supplement: Supplementary file 1 [file vaccines-12-00683-s001.zip › Supplementary table S3.pdf]

Supplementary Table S3. Adverse Reactions were stratified by age and time

| | 800L Batch1 (N=337) | | 800L Batch2 (N=335) | | 800L Batch3 (N=336) | | Total (N=1008) | | P |
|---|---|---|---|---|---|---|---|---|---|
| | n(%) | Frequency | n(%) | Frequency | n(%) | Frequency | n(%) | Frequency | |
| AR | 145(43.03) | 293 | 133(39.70) | 278 | 121(36.01) | 214 | 399(39.58) | 785 | 0.177 |
| AR within 30min | 25(7.42) | 31 | 15(4.48) | 16 | 15(4.46) | 16 | 55(5.46) | 63 | 0.151 |
| AR（0-7days） | 145(43.03) | 293 | 133(39.70) | 278 | 121(36.01) | 214 | 399(39.58) | 785 | 0.177 |
| AR（8-28days） | 0 | 0 | 0 | 0 | 0 | 0 | 0 | 0 | _ |
| 13-17 years old | | | | | | | | | |
| AR | 70(41.42) | 140 | 56(33.53) | 110 | 55(32.74) | 101 | 181(35.91) | 351 | 0.185 |
| AR within 30min | 17(10.06) | 18 | 13(7.78) | 13 | 11(6.55) | 11 | 41(8.13) | 42 | 0.489 |
| AR（0-7days） | 70(41.42) | 140 | 56(33.53) | 110 | 55(32.74) | 101 | 181(35.91) | 351 | 0.185 |
| AR（8-28days） | 0 | 0 | 0 | 0 | 0 | 0 | 0 | 0 | - |
| 6-12 years old | | | | | | | | | |
| AR | 75(44.64) | 153 | 77(45.83) | 168 | 66(39.29) | 113 | 218(43.25) | 434 | 0.435 |
| AR within 30min | 8(4.76) | 12 | 2(1.19) | 3 | 4(2.38) | 5 | 14(2.78) | 20 | 0.159 |
| AR（0-7days） | 75(44.64) | 153 | 77(45.83) | 168 | 66(39.29) | 113 | 218(43.25) | 434 | 0.435 |
| AR（8-28days） | 0 | 0 | 0 | 0 | 0 | 0 | 0 | 0 | - |

Adverse Reactions（AR）are adverse events related to vaccination by the judgment of the investigators. Subjects could have more than one adverse reaction. The p values are the comparisons among three batch groups. Comparisons among groups were made using the Chi-square Test or Fisher's Exact Test
