# Supplementary material for: Immunogenicity, Safety, and Immune Persistence of One Dose of SARS-CoV-2 Recombinant Adenovirus Type-5 Vectored Vaccine in Children and Adolescents Aged 6–17 Years: An Immunobridging Trial
Source: Vaccines (Basel). 2024 Jun 19;12(6):683. doi: 10.3390/vaccines12060683 (PMC11209179; doi:10.3390/vaccines12060683)
Supplement: Supplementary file 1 [file vaccines-12-00683-s001.zip › Supplementary table S4.pdf]

Supplementary Table S4. Local or systemic adverse reactions occurred within 7 days after the vaccination across three batch group （6-17 years old）

| | 800L Batch1 (N=337) | | 800L Batch2 (N=335) | | 800L Batch3 (N=336) | | Total (N=1008) | | P |
|---|---|---|---|---|---|---|---|---|---|
| | n(%) | Frequency | n(%) | Frequency | n(%) | Frequency | n(%) | Frequency | |
| Any | 145(43.03) | 293 | 133(39.70) | 278 | 121(36.01) | 214 | 399(39.58) | 785 | 0.177 |
| Local adverse reactions | 59(17.51) | 81 | 61(18.21) | 80 | 42(12.50) | 50 | 162(16.07) | 211 | 0.089 |
| Pain at the injection site | 57(16.91) | 57 | 58(17.31) | 58 | 40(11.90) | 40 | 155(15.38) | 155 | 0.096 |
| Swelling at the injection site | 7(2.08) | 7 | 7(2.09) | 7 | 2(0.60) | 2 | 16(1.59) | 16 | 0.204 |
| Induration at the injection site | 4(1.19) | 4 | 6(1.79) | 6 | 5(1.49) | 5 | 15(1.49) | 15 | 0.811 |
| Erythema at the injection site | 6(1.78) | 6 | 5(1.49) | 5 | 1(0.30) | 1 | 12(1.19) | 12 | 0.153 |
| Itching at the injection site | 6(1.78) | 6 | 3(0.90) | 3 | 2(0.60) | 2 | 11(1.09) | 11 | 0.383 |
| Rash | 1(0.30) | 1 | 1(0.30) | 1 | 0 | 0 | 2(0.20) | 2 | 0.777 |
| Systemic adverse reactions | 117(34.72) | 212 | 108(32.24) | 198 | 99(29.46) | 164 | 324(32.14) | 574 | 0.344 |
| Fever | 107(31.75) | 108 | 94(28.06) | 96 | 82(24.40) | 82 | 283(28.08) | 286 | 0.106 |
| Headache | 25(7.42) | 25 | 28(8.36) | 28 | 21(6.25) | 21 | 74(7.34) | 74 | 0.577 |
| Fatigue | 24(7.12) | 24 | 22(6.57) | 22 | 18(5.36) | 18 | 64(6.35) | 64 | 0.631 |
| Anorexia | 11(3.26) | 11 | 10(2.99) | 10 | 6(1.79) | 6 | 27(2.68) | 27 | 0.451 |
| Nausea | 10(2.97) | 10 | 9(2.69) | 9 | 7(2.08) | 7 | 26(2.58) | 26 | 0.761 |
| Vomit | 9(2.67) | 9 | 6(1.79) | 6 | 11(3.27) | 11 | 26(2.58) | 26 | 0.476 |
| Arthralgia | 7(2.08) | 7 | 4(1.19) | 4 | 2(0.60) | 2 | 13(1.29) | 13 | 0.248 |
| Oropharyngeal pain | 3(0.89) | 3 | 5(1.49) | 5 | 3(0.89) | 3 | 11(1.09) | 11 | 0.697 |
| Myalgia | 4(1.19) | 4 | 2(0.60) | 2 | 3(0.89) | 3 | 9(0.89) | 9 | 0.914 |
| Cough | 2(0.59) | 2 | 4(1.19) | 4 | 3(0.89) | 3 | 9(0.89) | 9 | 0.592 |
| Dizziness | 3(0.89) | 3 | 2(0.60) | 2 | 3(0.89) | 3 | 8(0.79) | 8 | >0.999 |
| Diarrhea | 1(0.30) | 1 | 2(0.60) | 2 | 2(0.60) | 2 | 5(0.50) | 5 | 0.752 |
| Abdominal pain | 1(0.30) | 1 | 0 | 0 | 2(0.60) | 2 | 3(0.30) | 3 | 0.665 |
| Chest discomfort | 0 | 0 | 1(0.30) | 1 | 1(0.30) | 1 | 2(0.20) | 2 | 0.554 |
| Pruritus | 1(0.30) | 1 | 1(0.30) | 1 | 0 | 0 | 2(0.20) | 2 | 0.777 |
| Hypersensitivity | 1(0.30) | 1 | 0 | 0 | 0 | 0 | 1(0.10) | 1 | >0.999 |
| Abdominal discomfort | 0 | 0 | 1(0.30) | 1 | 0 | 0 | 1(0.10) | 1 | 0.332 |
| Erythema | 1(0.30) | 1 | 0 | 0 | 0 | 0 | 1(0.10) | 1 | >0.999 |
| Dental ulcer | 1(0.30) | 1 | 0 | 0 | 0 | 0 | 1(0.10) | 1 | >0.999 |

| | | | | | | | | | |
|---|---|---|---|---|---|---|---|---|---|
| Sneezing | 0 | 0 | 1(0.30) | 1 | 0 | 0 | 1(0.10) | 1 | 0.332 |
| Insomnia | 0 | 0 | 1(0.30) | 1 | 0 | 0 | 1(0.10) | 1 | 0.332 |
| Dyspepsia | 0 | 0 | 1(0.30) | 1 | 0 | 0 | 1(0.10) | 1 | 0.332 |
| Congestion of the eye | 0 | 0 | 1(0.30) | 2 | 0 | 0 | 1(0.10) | 2 | 0.332 |

Any= all the participants with any adverse reactions. Adverse reactions are adverse events related to vaccination by the judgment of the investigators. Subjects could have more than one adverse reactions. The p values are the comparisons among three batch groups. Comparisons among groups were made using the Chi-square Test or Fisher's Exact Test
