# Supplementary material for: Immunogenicity, Safety, and Immune Persistence of One Dose of SARS-CoV-2 Recombinant Adenovirus Type-5 Vectored Vaccine in Children and Adolescents Aged 6–17 Years: An Immunobridging Trial
Source: Vaccines (Basel). 2024 Jun 19;12(6):683. doi: 10.3390/vaccines12060683 (PMC11209179; doi:10.3390/vaccines12060683)
Supplement: Supplementary file 1 [file vaccines-12-00683-s001.zip › Supplementary table S5.pdf]

Supplementary Table S5. Local or systemic adverse reactions occurred within 7 days after the vaccination across three batch group （13-17 years old）

	800L Batch1 (N=169)		800L Batch2 (N=167)		800L Batch3 (N=168)		Total (N=504)		P
	n(%)	Frequency	n(%)	Frequency	n(%)	Frequency	n(%)	Frequency	
Any	70(41.42)	140	56(33.53)	110	55(32.74)	101	181(35.91)	351	0.185
Local adverse reactions	34(20.12)	40	32(19.16)	38	28(16.67)	33	94(18.65)	111	0.703
Pain at the injection site	34(20.12)	34	32(19.16)	32	27(16.07)	27	93(18.45)	93	0.606
Induration at the injection site	0	0	2(1.20)	2	4(2.38)	4	6(1.19)	6	0.112
Itching at the injection site	3(1.78)	3	1(0.60)	1	1(0.60)	1	5(0.99)	5	0.627
Erythema at the injection site	2(1.18)	2	2(1.20)	2	0	0	4(0.79)	4	0.478
Swelling at the injection site	1(0.59)	1	1(0.60)	1	1(0.60)	1	3(0.60)	3	>0.999
Systemic adverse reactions	55(32.54)	100	38(22.75)	72	39(23.21)	68	132(26.19)	240	0.07
Fever	51(30.18)	52	29(17.37)	30	29(17.26)	29	109(21.63)	111	0.004
Fatigue	13(7.69)	13	10(5.99)	10	9(5.36)	9	32(6.35)	32	0.661
Headache	11(6.51)	11	12(7.19)	12	9(5.36)	9	32(6.35)	32	0.786
Nausea	3(1.78)	3	3(1.80)	3	4(2.38)	4	10(1.98)	10	0.927
Vomit	4(2.37)	4	1(0.60)	1	5(2.98)	5	10(1.98)	10	0.342
Anorexia	4(2.37)	4	3(1.80)	3	3(1.79)	3	10(1.98)	10	>0.999
Oropharyngeal pain	1(0.59)	1	4(2.40)	4	1(0.60)	1	6(1.19)	6	0.296
Arthralgia	3(1.78)	3	1(0.60)	1	1(0.60)	1	5(0.99)	5	0.627
Myalgia	3(1.78)	3	0	0	2(1.19)	2	5(0.99)	5	0.379
Dizziness	2(1.18)	2	1(0.60)	1	2(1.19)	2	5(0.99)	5	>0.999
Cough	1(0.59)	1	2(1.20)	2	1(0.60)	1	4(0.79)	4	0.701
Abdominal pain	1(0.59)	1	0	0	1(0.60)	1	2(0.40)	2	>0.999
Diarrhea	1(0.59)	1	1(0.60)	1	0	0	2(0.40)	2	0.776
Chest discomfort	0	0	1(0.60)	1	1(0.60)	1	2(0.40)	2	0.553
Sneezing	0	0	1(0.60)	1	0	0	1(0.20)	1	0.331
Insomnia	0	0	1(0.60)	1	0	0	1(0.20)	1	0.331
Dyspepsia	0	0	1(0.60)	1	0	0	1(0.20)	1	0.331
Pruritus	1(0.59)	1	0	0	0	0	1(0.20)	1	>0.999

Any= all the participants with any adverse reactions. Adverse reactions are adverse events related to vaccination by the judgment of the investigators. Subjects could have more than one adverse reactions. The p values are the comparisons among three batch groups. Comparisons among groups were made using the Chi-square Test or Fisher's Exact Test
