# Supplementary material for: Immunogenicity, Safety, and Immune Persistence of One Dose of SARS-CoV-2 Recombinant Adenovirus Type-5 Vectored Vaccine in Children and Adolescents Aged 6–17 Years: An Immunobridging Trial
Source: Vaccines (Basel). 2024 Jun 19;12(6):683. doi: 10.3390/vaccines12060683 (PMC11209179; doi:10.3390/vaccines12060683)
Supplement: Supplementary file 1 [file vaccines-12-00683-s001.zip › Supplementary table S6.pdf]

Supplementary Table S6. Local or systemic adverse reactions occurred within 7 days after the vaccination across three batch group （6-12 years old）

	800L Batch1 (N=168)		800L Batch2 (N=168)		800L Batch3 (N=168)		Total (N=504)		P
	n(%)	Frequency	n(%)	Frequency	n(%)	Frequency	n(%)	Frequency	
Any	75(44.64)	153	77(45.83)	168	66(39.29)	113	218(43.25)	434	0.435
Local adverse reactions	25(14.88)	41	29(17.26)	42	14(8.33)	17	68(13.49)	100	0.046
Pain at the injection site	23(13.69)	23	26(15.48)	26	13(7.74)	13	62(12.30)	62	0.078
Swelling at the injection site	6(3.57)	6	6(3.57)	6	1(0.60)	1	13(2.58)	13	0.117
Induration at the injection site	4(2.38)	4	4(2.38)	4	1(0.60)	1	9(1.79)	9	0.409
Erythema at the injection site	4(2.38)	4	3(1.79)	3	1(0.60)	1	8(1.59)	8	0.547
Itching at the injection site	3(1.79)	3	2(1.19)	2	1(0.60)	1	6(1.19)	6	0.875
Rash	1(0.60)	1	1(0.60)	1	0	0	2(0.40)	2	>0.999
Systemic adverse reactions	62(36.90)	112	70(41.67)	126	60(35.71)	96	192(38.10)	334	0.493
Fever	56(33.33)	56	65(38.69)	66	53(31.55)	53	174(34.52)	175	0.358
Headache	14(8.33)	14	16(9.52)	16	12(7.14)	12	42(8.33)	42	0.732
Fatigue	11(6.55)	11	12(7.14)	12	9(5.36)	9	32(6.35)	32	0.792
Anorexia	7(4.17)	7	7(4.17)	7	3(1.79)	3	17(3.37)	17	0.378
Nausea	7(4.17)	7	6(3.57)	6	3(1.79)	3	16(3.17)	16	0.432
Vomit	5(2.98)	5	5(2.98)	5	6(3.57)	6	16(3.17)	16	0.937
Arthralgia	4(2.38)	4	3(1.79)	3	1(0.60)	1	8(1.59)	8	0.547
Cough	1(0.60)	1	2(1.19)	2	2(1.19)	2	5(0.99)	5	>0.999
Oropharyngeal pain	2(1.19)	2	1(0.60)	1	2(1.19)	2	5(0.99)	5	>0.999
Myalgia	1(0.60)	1	2(1.19)	2	1(0.60)	1	4(0.79)	4	>0.999
Diarrhea	0	0	1(0.60)	1	2(1.19)	2	3(0.60)	3	0.776
Dizziness	1(0.60)	1	1(0.60)	1	1(0.60)	1	3(0.60)	3	>0.999
Hypersensitivity	1(0.60)	1	0	0	0	0	1(0.20)	1	>0.999
Abdominal discomfort	0	0	1(0.60)	1	0	0	1(0.20)	1	>0.999
Abdominal pain	0	0	0	0	1(0.60)	1	1(0.20)	1	>0.999
Erythema	1(0.60)	1	0	0	0	0	1(0.20)	1	>0.999
Dental ulcer	1(0.60)	1	0	0	0	0	1(0.20)	1	>0.999
Congestion of the eye	0	0	1(0.60)	2	0	0	1(0.20)	2	>0.999
Pruritus	0	0	1(0.60)	1	0	0	1(0.20)	1	>0.999

Any= all the participants with any adverse reactions. Adverse reactions are adverse events related

to vaccination by the judgment of the investigators. Subjects could have more than one adverse reactions. The p values are the comparisons among three batch groups. Comparisons among groups were made using the Chi-square Test or Fisher's Exact Test
